# Characteristics of equipartition for RNA structure

**DOI:** 10.1186/1753-6561-8-S6-S3

**Published:** 2014-10-13

**Authors:** Hengwu Li, Daming Zhu, Caiming Zhang, Huijian Han, Keith A Crandall

**Affiliations:** 1 School of Computer Science and Technology, and Shandong Provincial Key Laboratory of Digital Media Technology, Shandong University of Finance and Economics, Jinan 250014, China; 2 School of Computer Science and Technology, and Shandong Provincial Key Laboratory of Software Engineering, Shandong University, Jinan 250014, China; 3 Computational Biology Institute, George Washington University, Ashburn, Virginia 20147, USA

## Abstract

**Background:**

With the continuous discovery of novel RNA molecules with key cellular functions and of novel pathways and interaction networks, the need for structural information of RNA is still increasing. In order to predict structure of long RNA and understand its natural folding mechanism, exploring the characteristic of RNA structure is an important issue.

**Methods:**

The real RNA secondary structures of all 480 sequences from the database of RNA strand, validated by nuclear magnetic resonance or x-ray are selected. For one sequence with multiple domains, the length ratios of these domains to the sequence are computed. For one sequence with one domain and multiple sub-domains, the length ratios of these sub-domains to the domain are computed. Then the ratios are compared and analyzed to seek the partition characteristic of domains and subdomains.

**Results:**

For most RNAs, the length ratios of multiple domains to its sequence are close to equal, and those of sub-domains to its domain are also nearly identical. Most RNAs with multiple domains have two domains, so the length ratios of the domains to its sequence are close to 0.5. For sequence with one domain and no sub-domain or one sub-domain, the centre of domain and sub-domain is close to that of the sequence.

**Conclusions:**

A novel finding is given that RNA folding accords with the characteristic of equipartition based on statistical analysis. The characteristic reflects the folding rules of RNA from a new angle, which maybe more close to natural folding.

## Background

RNAs are versatile molecules. To understand fully the various functions of RNAs, we need to first understand their structures [1]. Experimental test of RNA tertiary structure is too expensive and time consuming to meet practical need, so predicting RNA structure by computer becomes a basic method and issue in computational biology [2].

RNA is folded as the process of transcription into RNA from DNA. In order to predict RNA structure, a case may be made that the natural folding process of RNA and the simulated folding of RNA using an evolutionary algorithm, which includes intermediate folds, have much in common [3]. So exploring the characteristic of RNA structure is an important issue to understand its natural folding mechanism.

We compare the structures of the test set of all 480 sequences from RNA STRAND [4], validated by NMR or X-Ray, and give a novel finding that RNA folding accords with characteristic of equipartition based on statistical analysis on real RNA secondary structures.

## Methods

Let sequence *s*=*s*_1_*s*_2_*...s_n _*be a single-stranded RNA molecule, where each base $s_{i}\in \left\{A,U,C,G\right\}$, 1 ≤ *i *≤ *n*. The subsequence *s_i, j _*= *s*_*i *_*s*_*i*+1_. . . *s_j _*is a segment of *s*, 1 ≤ *i *≤ *j *≤ *n*.

If *s_i _*and *s_j _*are complementary bases (*A*&*U, C*&G, *U*&*G*), then *s_i _*and *s_j _*may constitute a base pair (*i, j*). A secondary structure *S *on *s *is a set of base pairs *S*={(*i, j*)}, where i,j∈{1,2,⋯,n}, that satisfies the following conditions.

(*No sharp turns*.) The ends of each pair in *S *are separated by at least four intervening bases; that is, if (i,j)∈S, then *i < j-*3.

For any pair (*i, j*) in S,(i,j)∈{(A,U),(C,G),(U,G),(U,A),(G,C),(G,U)}.

*S *is a matching: no base appears in more than one pair.

(*The non-crossing condition*.) If (*i, j*) and (*k, l*) are two pairs in *S*, then they are compatible, that is, they are juxtaposed (e.g. *i *<*j < k *<*l*) or nested (e.g. *i *<*k *<*l *<*j*).

If (*i, j*) and (i+1,j-1)∈S, base pairs (*i, j*) and (*i+*1, *j-*1) constitute stack (*i, i+*1: *j-*1*, j*), and *m*(≥1) consecutive stacks form the helix (*i, i+m*: *j-m, j*) with the length of *m*+1.

If base pairs (*i, j*) and (*k, l*) are incompatible, they form a pseudoknot (e.g. *i < k < j < l*). More complex pseudoknots may occur if three or more base pairs cross each other.

In the past domains have been described as units of: compact three-dimensional structure, folding, function and evolution [5]. A domain is a conserved part of a given sequence and structure that exists independently of the rest of the chain, and often can be independently stable and folded. The majority of domains have less than 200 residues with an average of approximately 100 residues [6].

A domain *D(i', j') *consists of all (*i*', *j*') that satisfy, (*i*', *j') ∈ *D(*i, j*) then *i < i' < j' < j*. Each base pair and each helix is placed uniquely in one domain [7].

**Figure 1:**
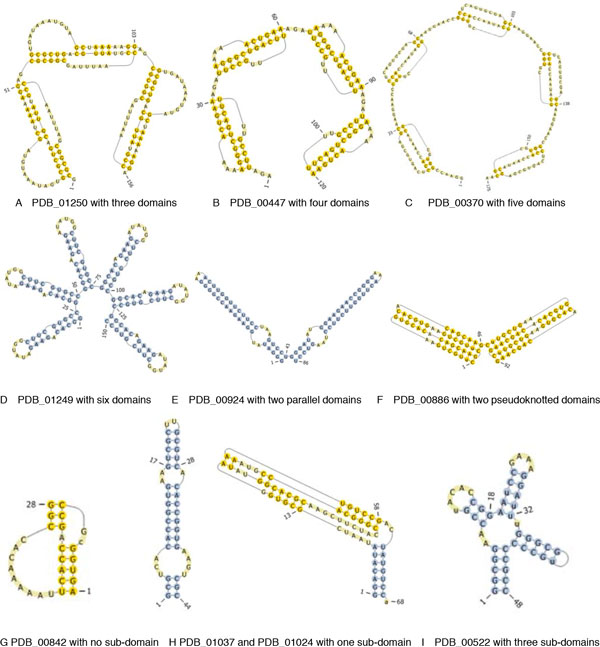
**Structures of different domains and sub-domains**. (a) Structure of three domains. (b) Structure of four domains. (c) Structure of five domains. (d) Structure of six domains. (e) Structure of two parallel domains formed by helixes. (f) Structure of two parallel domains formed by pseudokonts. (g) Structure of one domain with no sub-domains. (h) Structure of one domain with one sub-domain. (i) Structure of one domain with multiple sub-domains.

A domain is closed by a helix or pseudoknot, as Figure 1. One sub-domain is an independently stable part of one domain. If the closed helix or pseudoknot of one domain is deleted, its sub-domain will become domain.

By convention, single strands of RNA sequences are written in 5'-to-3' direction. RNA is folded as the process of transcription into RNA from DNA. The subsequence *s_i,j _*begins to transcribe from the 5'-end *s_i_*. It terminates transcription at the 3'-end *s_j_*, as Figure 1. The helix (*i, i*+*m*: *j-m, j*) is totally folded after transcription of *s_j_*.

For purpose of understanding the natural folding mechanism and pathway of RNA, we selected the real structures of all 480 sequences from RNA STRAND with secondary and pseudoknotted structures, validated by NMR or X-Ray, non-fragment and non-redundant sequences, and analyzed their domains and sub-domains.

If the structure of RNA has multiple domains, we computed *R *as the ratios of 3'-end of domains to the length of sequence. Let *L *is the length of sequence. The ratio of the 3'-end of the helix (*i, i*+*m*: *j-m, j*) and the domain *D*(*i, j*) to the length of sequence *s*_1,*L *_is the ratio of *j *to *L*, that is *R*=*j*/*L*. We compute and analyze the value of *R*, and seek the partition characteristic of domains.

If the structure of RNA has only one domain, we computed *SR *as the length ratios of its sub-domains to the domain. If the domain *D*(*i, j*) is closed by a helix (*i, i*+*m*: *j-m, j*), then its internal length is *j-i-2m-1*, and *SR *is equal to (*q-p+1*)/(*j-i-2m-1*) for the sub-domain *D(i+p, i+q) *with *j-m-i>q>p>m*. We compute and analyze the value of *SR*, and seek the partition characteristic of sub-domains.

## Results and discussion

### Characteristic of equipartition for synthetic RNA

**Figure 2:**
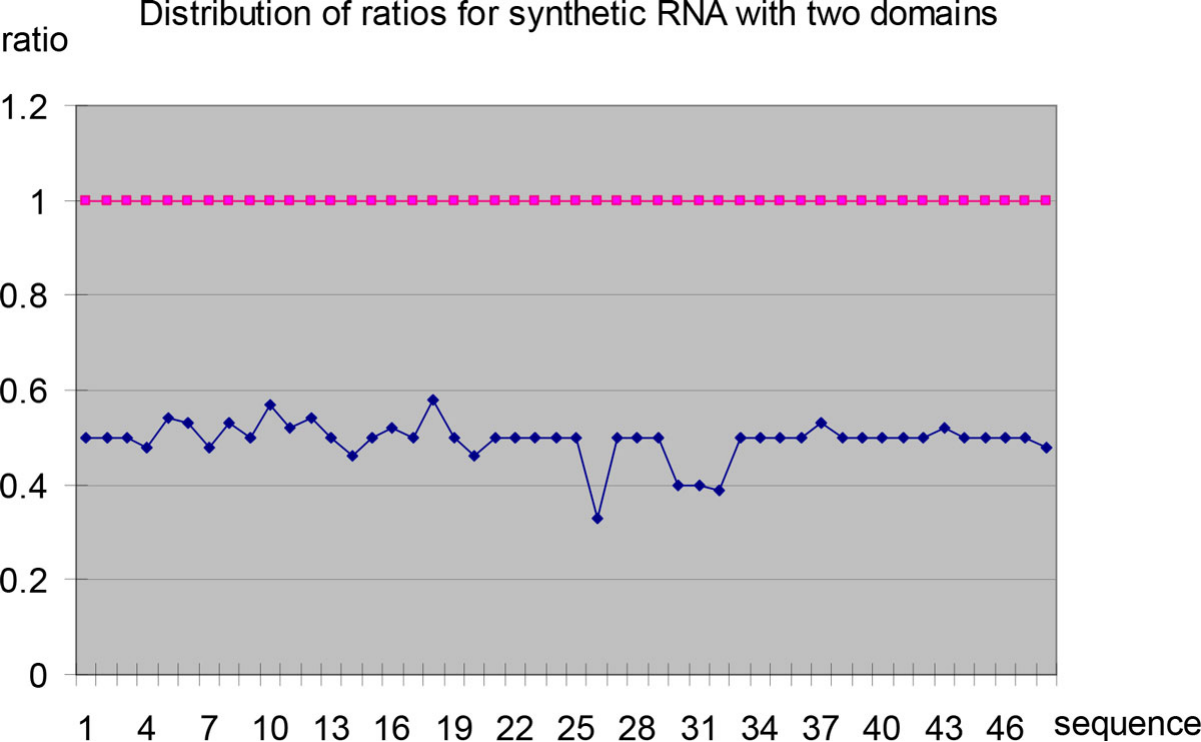
**Distribution of ratios for synthetic RNA with two domains**. (a) For all validated by NMR or X-Ray, non-fragment and non-redundant 48 synthetic RNA sequences with two domains from RNA STRAND, the length ratio of 3'-end of domains to its sequence is computed and the summarization is shown. The first ratio centres on 0.5, and the second ratio is 1.0. (b) The x-axis represents the sequence, and the y-axis represents length ratio of the 3'-end of domain to the sequence.

We compare the structures of all 248 sequences of synthetic RNA. The results of statistical analysis on these structures are shown in Figure 1, Figure 2, Table 1 Table 2 and Table 3.

**Table 1:** Distribution of domains for synthetic RNA with more than two domains

Sequences	L	D 1	D 2	D 3	D 4	D 5	D 6	R 1	R 2	R 3	R4	R 5	R 6
PDB_00195	84	1-28	29-56	57-84				0.33	0.66	1.0			
PDB_00262	48	1-16	17-32	33-48				0.33	0.66	1.0			
PDB_00754	55	1-17	18-37	38-54				0.32	0.69	1.0			
PDB_01250	156	15-52	53-104	105-156				0.32	0.69	1.0			
PDB_01060	64		17-32	33-48	49-64				0.5	0.75	1.0		
PDB_00175	96	1-24	25-48	49-64	65-96			0.25	0.5	0.75	1.0		
PDB_00873	96	1-24	25-48	49-64	65-96			0.25	0.5	0.75	1.0		
PDB_00447	120	1-30	41-60	71-90	101-120			0.25	0.5	0.75	1.0		
PDB_00340	140	1-35	36-70	71-105	106-140			0.25	0.5	0.75	1.0		
PDB_01061	80	1-16	33-48	49-64	65-80			0.2	0.4	0.6	0.8	1.0	
PDB_00370	175	1-35	36-70	71-105	106-140	141-175		0.2	0.4	0.6	0.8	1.0	
PDB_01249	150	1-25	26-50	51-75	76-100	101-125	126-150	0.17	0.33	0.5	0.67	0.83	1.0

L is the length of the sequence, D1-D6 are the domains of the sequence, R1-R6 are the ratios of 3'-end of domains to the length of sequence. The domain is expressed as the 5'-end and 3'-end.

**Table 2:** Distribution of domains for synthetic RNA with two domains

sequences	L	H1	H2	D1	D2	R 1	R2	sequences	L	H1	H2	D1	D2	R 1	R2
PDB_00483	58	1-29	30-58	1-29	30-58	0.5	1.0	PDB_00123	92	1-46	49-92	1-46	47-92	0.5	1.0
PDB_00816	46	1-23	24-46	1-23	24-46	0.5	1.0	PDB_00196	57	1-18	20-57	1-19	20-57	0.33	1.0
PDB_00924	86	1-43	44-86	1-43	44-86	0.5	1.0	PDB_00236	48	1-24	25-48	1-24	25-48	0.5	1.0
PDB_00942	31	1-15	17-31	1-15	16-31	0.48	1.0	PDB_00254	24	1-12	13-24	1-12	13-24	0.5	1.0
PDB_00963	28	1-15	16-28	1-15	16-28	0.54	1.0	PDB_00264	48	1-24	26-48	1-24	25-48	0.5	1.0
PDB_00964	36	1-19	22-34	1-19	20-36	0.53	1.0	PDB_00709	40	1-16	17-32	1-16	17-40	0.4	1.0
PDB_00965	21	2-10	13-21	1-11	12-21	0.52	1.0	PDB_00710	40	1-16	17-32	1-16	17-40	0.4	1.0
PDB_00966	23	2-10	13-23	1-12	13-23	0.52	1.0	PDB_00868	52	1-19	21-39	1-20	21-52	0.39	1.0
PDB_00970	34	1-17	20-32	1-17	18-34	0.5	1.0	PDB_00663	44	2-19	26-41	1-22	23-44	0.5	1.0
PDB_00971	30	1-17	18-30	1-17	18-30	0.57	1.0	PDB_00682	32	1-16	17-32	1-16	17-32	0.5	1.0
PDB_00973	27	2-14	15-27	1-14	15-27	0.52	1.0	PDB_00688	40	1-20	21-40	1-20	21-40	0.5	1.0
PDB_00974	28	1-15	16-28	1-15	16-28	0.54	1.0	PDB_00724	66	1-32	34-65	1-33	34-66	0.5	1.0
PDB_00979	154	3-77	80-154	1-77	78-154	0.5	1.0	PDB_00866	19	2-9	11-19	1-10	11-19	0.53	1.0
PDB_01035	56	1-26	27-56	1-26	27-56	0.46	1.0	PDB_00871	48	1-24	25-48	1-24	25-48	0.5	1.0
PDB_01130	60	1-30	31-60	1-30	31-60	0.5	1.0	PDB_00874	44	1-20	23-42	1-22	23-44	0.5	1.0
PDB_01135	29	1-15	17-29	1-15	17-29	0.52	1.0	PDB_00886	92	1-46	47-92	1-46	47-92	0.5	1.0
PDB_01136	30	2-14	17-29	2-14	15-29	0.5	1.0	PDB_00892	72	1-36	48-61	1-36	37-72	0.5	1.0
PDB_01138	26	1-15	18-25	1-15	16-26	0.58	1.0	PDB_00929	40	1-20	21-40	1-20	21-40	0.5	1.0
PDB_01145	40	1-20	21-40	1-20	21-40	0.5	1.0	PDB_00960	29	2-13	18-29	2-15	16-29	0.52	1.0
PDB_01164	56	1-26	27-56	1-26	27-56	0.46	1.0	PDB_01017	30	2-15	17-30	2-15	16-30	0.5	1.0
NDB_00010	8	1-4	5-8	1-4	5-8	0.5	1.0	PDB_01019	30	2-15	17-30	2-15	16-30	0.5	1.0
NDB_00037	8	1-4	5-8	1-4	5-8	0.5	1.0	PDB_01156	88	1-43	45-87	1-44	45-88	0.5	1.0
NDB_00048	56	1-28	29-56	1-28	29-56	0.5	1.0	PDB_01219	44	1-21	23-44	1-22	23-44	0.5	1.0
PDB_00104	36	1-18	19-36	1-18	19-36	0.5	1.0	PDB_00945	31	2-14	15-30	1-14	15-31	0.48	1.0

L is the length of the sequence, H1-H2 are the helixes of the sequence, D1-D2 are the domains of the sequence, R1-R2 are the ratios of 3'-end of domains to the length of sequence. The helix data is the closed base pair in helix or the start and end bases in pseudokont. The domain is expressed as 5'-end and 3'-end.

**Table 3:** Distribution of multiple sub-domains for synthetic RNA with one domain

Sequences	L	H1	H2	H3	SD1	SD2	SD3	D	R 1	R 2	R 3
PDB_01111	75	7-38	40-68		7-38	39-68		7-68	0.52	0.48	
PDB_01112	75	7-39	41-68		7-39	40-68		7-68	0.53	0.47	
PDB_01114	76	7-39	41-69		7-39	41-69		7-69	0.52	0.48	
PDB_01115	74	7-36	40-66		7-36	37-67		7-67	0.49	0.51	
PDB_01116	69	7-37	39-62		7-37	38-62		7-62	0.55	0.45	
PDB_00522	48	8-18	19-32	34-43	6-18	19-32	33-44	6-44	0.33	0.36	0.31
PDB_00750	155	5-19	21-32	35-151	5-20	21-34	35-151	5-151	0.11	0.1	0.79

L is the length of the sequence, H1-H3 are the helixes of the sequence, D is the domain of the sequence, SD1-SD3 are the sub-domains of the domain, R1-R3 are the length ratios of SD1-SD3 to D. The helix data is the closed base pair or the start and end bases in pseudokont. The domain is expressed as 5'-end and 3'-end of the internal section except of closed helix or pseudokont.

Table 1 shows the distribution of multi-domains for synthetic RNA with more than two domains.

There are 12 sequences with more than two domains as Table 1 and Figure 1. Sequences PDB_00195, PDB_00262, PDB_00754 and PDB_01250, have three domains. Their domains are 0-0.33*L*, 0.33*L*-0.66*L *and 0.66*L-L *as Table 1 and Figure 1A, which completely fits the characteristic of equipartition. Sequence PDB_01060 has three domains, 0.25*L*-0.5*L*, 0.5*L*-0.75*L *and 0.75*L-L*, but we can divide the sequence into four domains, 0-0.25*L*, 0.25*L*-0.5*L*, 0.5*L*-0.75*L *and 0.75 *L-L*, then it also conforms to the characteristic of equipartition.

Sequences PDB_00175, PDB_00873, PDB_00447 and PDB_00340, have four domains. Their domains are 0-0.25*L*, 0.25*L*-0.5*L*, 0.5*L*-0.75*L *and 0.75*L*- *L *as Table 1 and Figure 1B, which completely fits the characteristic of equipartition.

Sequences PDB_01061 and PDB_00370 have five domains. Their domains are 0-0.2*L*, 0.2*L*-0.4*L*, 0.4*L*-0.6*L*, 0.6 *L*-0.8*L *and 0.8*L-L *as Table 1 and Figure 1C, which completely fits the characteristic of equipartition.

Only sequence PDB_01249 has six domains 0-0.17*L*, 0.17*L*-0.33*L*, 0.33*L*-0.5*L*, 0.5*L*-0.67*L*, 0.67*L*-0.83*L *and 0.83*L-L*, which is completely fits the characteristic of equipartition, as Table 1 and Figure 1D.

Table 2 shows the distribution of domains for synthetic RNA with two domains. There are 48 sequences with two domains as Table 2 and Figure 2. The domains of 29 sequences are just 0-0.5*L *and 0.5*L-L*, and those of 13 sequences are close to 0-0.5*L *and 0.5*L-L*, which fits the characteristic of equipartition. The domain is formed by parallel helixes or pseudoknots as Figure 1E and 1F. But there are some exceptions, the domains of sequence PDB_00196 are 0-0.33*L *and 0.33*L-L*, those of sequence PDB_00868 are 0-0.39*L *and 0.39*L-L*, those of sequence PDB_00709 and PDB_00710 are 0-0.4*L *and 0.4*L-L*, those of sequence PDB_00971 are 0-0.57*L *and 0.57*L-L*, and those of sequence PDB_01138 are 0-0.58*L *and 0.58*L-L*. It can be thought as the combination of some domains, and they close to 0.33*L*, 0.4*L *and 0.6*L*. For example, we can regard sequence PDB_00196 as three domains 0-0.33*L*, 0.33*L- *0.66*L *and 0.66*L-L*, then 0.33*L-*0.66*L *and 0.66*L-L *combines into domain 0.33*L-L*.

The rest of 188 sequences have one domain or one pseudokont, and the centre of domain is basically same as that of its sequence. In common, their sub-domains can be divided into three classes, no sub-domain in 157 sequences as Figure 1G, one subdomain with the centre is close to that of its sequence as Figure 1H, and multiple and nearly equal sub-domains as Figure 1I.

For Sequence with one domain and no sub-domain, the centre of domain is close to that of the sequence. There are 11 sequences with one pseudoknotted domain. Sequences PDB_01040, PDB_00020, PDB_01165 and PDB_01194 have one pseudoknot with three helixes, and the helix (2,8:18,24) and (26,32:42,48) in PDB_01040, (1,5:15,21) and (22,27:35,40) in PDB_00020, (1,9:12,20) and (23,31:34,42) in PDB_01165, (1,5:12,16) and (17,21:28,32) in PDB_01194, meet the characteristic of equipartition. PDB_00053, PDB_00209, PDB_00134, PDB_00842, PDB_01059 and PDB_00124 have one pseudoknot with two helixes, and the centre of the pseudoknot is same as that of its sequence. PDB_00759 has two pseudoknots with three helixes, and the centre of helix (3,4:13,14) is basically same as that of its sequence.

As Table 3 shows the distribution of sub-domains for synthetic RNA with one domain and multiple sub-domains. There are 7 sequences with one domain and multiple subdomains, as Table 5. The sub-domains of six sequences also conform to the characteristic of equipartition, with only one sequence exception.

**Table 4:** Distribution of domains for tRNA with multiple domains

Sequence ID	L	N	D1	D2	D3	D4	D5	R1	R2	R3	R4	R5
PDB_00307	150	2	1-75	76-150				0.5	1.0			
PDB_00421	152	2	1-75	76-152				0.49	1.0			
PDB_00472	147	2	1-73	74-147				0.5	1.0			
PDB_00475	148	2	1-74	75-148				0.5	1.0			
PDB_00593	152	2	1-75	76-152				0.5	1.0			
PDB_00648	42	2	1-20	21-42				0.48	1.0			
PDB_00649	39	2	1-21	22-39				0.54	1.0			
PDB_00681	51	2	1-17	18-51				0.33	1.0			
PDB_00722	152	2	1-76	77-152				0.5	1.0			
PDB_00891	44	2	1-23	24-44				0.52	1.0			
PDB_00904	152	2	1-76	77-152				0.5	1.0			
PDB_00980	150	2	1-75	76-150				0.5	1.0			
PDB_00981	150	2	1-74	75-150				0.49	1.0			
PDB_00994	146	2	1-72	73-146				0.49	1.0			
PDB_01054	154	2	2-77	78-154				0.5	1.0			
PDB_01074	75	2	1-37	38-75				0.49	1.0			
PDB_01162	56	2	1-34	35-56				0.61	1.0			
PDB_00637	69	3	1-26	27-46	47-69			0.38	0.67	1.0		
PDB_00732	78	3	1-25	26-51	52-78			0.32	0.65	1.0		
PDB_00733	77	3	1-26	27-51	52-77			0.34	0.66	1.0		
PDB_00998	145	4	1-26	27-49	50-73	74-145		0.18	0.34	0.5	1.0	
PDB_00398	380	5	1-76	77-152	153-228	229-304	305-380	0.2	0.4	0.6	0.8	1.0
PDB_01000	148	6	1-26	27-50	51-74	75-100	101-124	0.18	0.34	0.5	0.68	0.83
							125-148					1.0

L is the length of the sequence, N is the number of domains in the sequence, D1-D5 are the domains of the sequence, R1-R5 are the ratios of 3'-end of domains to the length of sequence. The domain is expressed as 5'-end and 3'-end.

**Table 5:** Distribution of sub-domains for tRNA with one domain and multiple sub-domains

Sequences	L	H1	H2	H3	SD1	SD2	SD3	D	R 1	R 2	R 3
NDB_00051	75	10-25	26-44	48-64	8-25	26-44	45-64	8-64	0.32	0.33	0.35
PDB_00045	76	10-25	26-44	48-65	8-25	26-45	46-65	8-65	0.31	0.34	0.34
PDB_00070	76	10-25	26-44	48-65	8-25	26-45	46-65	8-65	0.31	0.34	0.34
PDB_00095	75	10-25	26-43	48-64	8-25	26-44	45-64	8-64	0.32	0.33	0.35
PDB_00229	75	10-24	25-43	48-64	8-24	25-44	45-64	8-64	0.30	0.35	0.35
PDB_00244	73	10-25	26-44	48-64	8-25	26-44	45-64	8-64	0.32	0.33	0.35
PDB_00259	72	10-25	26-44	48-64	8-25	26-44	45-64	8-64	0.32	0.33	0.35
PDB_00313	74	10-24	26-42	47-63	8-25	26-44	45-63	8-63	0.32	0.34	0.34
PDB_00376	73	9-23	24-42	46-62	7-23	24-42	43-62	7-62	0.30	0.34	0.36
PDB_00426	74	9-23	24-41	47-63	7-23	24-43	44-63	7-63	0.30	0.35	0.35
PDB_00903	76	10-25	26-44	49-65	8-25	26-45	46-65	8-65	0.31	0.34	0.34
PDB_00999	70	10-26	27-45	50-64	5-26	27-47	48-67	5-67	0.35	0.33	0.32

L is the length of the sequence, H1-H3 are the helixes of the sequence, D is the domain of the sequence, SD1-SD3 are the sub-domains of the domain, R1-R3 are the length ratios of SD1-SD3 to D. The helix data is the closed base pair or the start and end bases in pseudokont. The domain is expressed as 5'-end and 3'-end of the internal section except of closed helix or pseudokont.

### Characteristic of equipartition for tRNA

We compare the structures of all 46 sequences of tRNA. The results of statistical analysis on these secondary structures are shown in Table 4 and Table 5.

Table 4 shows the distribution of domains for tRNA with multiple domains. There are 17 sequences with two domains. The domains of 8 sequences are just 0-0.5*L *and 0.5*L-L*, and those of 7 sequences are close to 0-0.5*L *and 0.5*L-L*, which fits the characteristic of equipartition. But there are some exceptions, the domains of sequence PDB_00681 are 0-0.33*L *and 0.33*L-L*, those of sequence PDB_01162 are 0-0.61*L *and 0.61*L-L*. It can also be thought as the combination of two domains, and they close to 0.33*L *and 0.66*L*.

There are 3 sequences have three domains. Their domains are close to 0-0.33*L*, 0.33*L *-0.66*L *and 0.66*L-L*, which fits the characteristic of equipartition.

Sequence PDB_00998 has four domains 0-0.18*L*, 0.18*L*-0.34*L*, 0.34*L*-0.5*L *and 0.5*L-L*. They can be thought as two groups, one is 0-0.5*L*, and the other is 0.5*L-L*, which fits the characteristic of equipartition. For the group 0-0.5*L*, it is divided into three domains, 0-0.18*L*, 0.18*L*-0.34*L *and 0.34*L*-0.5*L*, it also fits the characteristic.

Sequence PDB_00398 has five domains 0-0.2*L*, 0.2*L*-0.4*L*, 0.4*L*-0.6*L*, 0.6*L*-0.8*L *and 0.8*L-L*, which completely fits the characteristic of equipartition.

Sequence PDB_01000 has six domains 0-0.18 *L*, 0.18 *L*-0.34*L*, 0.34*L *-0.5*L*, 0.5*L*-0.68 *L*, 0.68*L*-0.83*L *and 0.83*L-L*, which is also fits the characteristic of equipartition.

The rest of 23 sequences have one domain or one pseudokont, and the centre of domain is basically same as that of its sequence. There are 12 sequences with one domain and multiple sub-domains as Table 5 and their sub-domains all close to 0 - 0.33*L*, 0.33*L*-0.66*L *and 0.66*L-L*, which conforms to the characteristic of equipartition.

### Characteristic of equipartition for other RNA

We compare the structures of all 49 sequences of Other RNA, 6 sequences of Ham Ribozyme and 9 sequences of Viral & Phag.

The results of statistical analysis on these secondary structures are shown in Table 6. For Other RNA, there are 7 sequences with two domains, and the domains are just 0-0.5*L *and 0.5*L-L*, which completely fits the characteristic of equipartition. There are 3 sequences have three domains. The domains of PDB_00626 and PDB_00739 are just 0-0.33*L*, 0.33*L*-0.67*L *and 0.67*L-L*, which fits the characteristic of equipartition. Sequence PDB_01261 has three domains. They can be divided into two groups, one is 0-0.5*L*, and the other is 0.5*L-L*. Then the domain 0.5*L-L *is divided into 0.5*L*-0.75*L *and 0.75*L-L*. Sequence PDB_001261 and PDB_00985 has four domains. Their domains are close to 0-0.25*L*, 0.25*L*-0.5*L*, 0.5-0.75*L *and 0.75*L-L*, which fits the characteristic of equipartition. Sequences PDB_01061 and PDB_00370 have five domains. Their domains are 0-0.33*L*, 0.33*L*-0.66*L *and 0.66*L-L*, which completely fits the characteristic of equipartition.

**Table 6:** Distribution of domains for Other RNA, Viral Phage and Ham Ribozyme with multiple domains

Sequence ID	Type	L	N	D1	D2	D3	D4	R1	R2	R3	R4
PDB_00308	Other RNA	150	2	1-75	76-150			0.5	1.0		
PDB_00358	Other RNA	146	2	1-73	74-146			0.5	1.0		
PDB_00419	Other RNA	150	2	1-75	76-150			0.5	1.0		
PDB_00804	Other RNA	176	2	1-88	89-176			0.5	1.0		
PDB_00967	Other RNA	156	2	1-78	79-156			0.5	1.0		
PDB_00983	Other RNA	152	2	1-75	76-152			0.5	1.0		
PDB_01274	Other RNA	152	2	1-76	77-152			0.5	1.0		
PDB_00626	Other RNA	225	3	1-75	76-150	151-225		0.33	0.67	1.0	
PDB_00739	Other RNA	228	3	1-76	77-152	153-228		0.33	0.67	1.0	
PDB_01261	Other RNA	968	3	1-484	485-726	727-968		0.5	0.75	1.0	
PDB_00985	Other RNA	248	4	1-62	63-124	125-186	187-248	0.25	0.5	0.75	1.0
PDB_01161	Other RNA	272	4	1-72	73-144	145-210	211-272	0.26	0.52	0.77	1.0
PDB_00743	Viral Phage	33	2	1-17	18-33			0.52	1.0		
PDB_00157	Ham. Ribozyme	82	2	3-39	44-80	1-41	42-82	0.5	1.0		

L is the length of the sequence, N is the number of domain in the sequence, D1-D4 are the domains of the sequence, R1-R4 are the ratios of 3'-end of domains to the length of sequence. The domain is expressed as 5'-end and 3'-end.

For Viral & Phage, only one sequence PDB_00743 has two domains 0-0.52*L*, 0.52*LL*, which fits the characteristic of equipartition, as Table 6. The rest of 8 sequences have only one domain and no sub-domain, and three of them exist as two pseudoknotted helixes. The domains all fit the characteristic of equipartition.

For Ham Ribozyme, only one sequence PDB_00157 has two domains 0-0.5*L*, 0.5*L-L*, which completely fits the characteristic of equipartition, as Table 6. The rest of 5 sequences only have one domain with two sub-domains, and their sub-domains also conform to the characteristic of equipartition.

### Characteristic of equipartition for other ribozyme

We compare the structures of all 18 sequences of Other Ribozyme. The results of statistical analysis on these secondary structures are shown in Table 7.

**Table 7:** Distribution of domains for Other Ribozyme

Sequence ID	L	N	D1	D 2	D3	D4	R1	R2	R3	R4
PDB_00078	316	1	1-115	116-151	152-273	1-311	0.36	0.48	1.0	
PDB_00851	98	2	1-49	50-98			0.5	1.0		
PDB_00856	98	2	1-49	50-98			0.5	1.0		
PDB_00893	61	2	1-25	26-61			0.41	1.0		
PDB_00956	61	2	1-25	26-61			0.41	1.0		
PDB_01068	142	2	1-71	72-142			0.5	1.0		
PDB_01069	141	2	1-70	71-141			0.5	1.0		
PDB_01092	143	2	1-72	73-143			0.5	1.0		
PDB_01255	159	2	1-112	113-159			0.7	1.0		
PDB_01300	142	2	4-72	73-142			0.5	1.0		
PDB_01301	141	2	2-70	71-141			0.5	1.0		
PDB_01302	139	2	2-70	71-138			0.5	1.0		
PDB_00176	96	4	2-24	26-48	50-72	74-96	0.25	0.5	0.75	1.0
PDB_01187	142	4	1-51	52-71	72-121	124-142	0.36	0.5	0.85	1.0
PDB_00805	968	8	1-242	243-484	485-726	727-968	0.25	0.5	0.75	1.0

L is the length of the sequence, N is the number of domain in the sequence, D1-D4 are the domains of the sequence, R1-R4 are the ratios of 3'-end of domains to the length of sequence. The domain is expressed as 5'-end and 3'-end.

Sequence PDB_00805 have 8 domains, and they can be divided into four groups 0-0.25*L*, 0.25*L*-0.5*L*, 0.5-0.75*L *and 0.75*L-L*, which conforms to the characteristic of equipartition. Sequence PDB_00176 has four domains, and they meet the character of equipartition. Sequence PDB_01187 has four domains, and they can be divided into two groups 0-0.5*L *and 0.5*L-L*, which conforms to the characteristic of equipartition. There are 11 sequences with two domains. They fit the character of equipartition with three exceptions.

There are 4 sequences have only one domain. PDB_00088 has one domain with no sub-domain, PDB_00142 has one domain with one sub-domain, and they also fit the characteristic of equipartition. Sequence PDB_00078 has one pseudokontted domain with four sub-domains 0-0.36*L*, 0.36*L-*0.48*L*, 0.48*L-*0.86*L *and 0.86*L-L*. They can be divided into two groups, one is 0-0.48*L*, and the other is 0.48*L-L*, which nearly fit the characteristic of equipartition. PDB_01185 has one pseudokontted domain with three sub-domains, and sub-domain 0-0.52*L *and 0.52*L-L *nearly fit the characteristic of equipartition.

For 16S rRNA and 32S rRNA, they conform to other characteristics besides the characteristic of equipartition, it is a matter for further discussion.

## Conclusions

In this paper, we give a novel finding that RNA folding accords with the characteristic equipartition based on statistical analysis on real RNA secondary structures of all 480 sequences from RNA STRAND, validated by NMR or X-Ray. For most RNA sequences, the length of multiple domains is close to equal. For the sub-domains of one domain, the length of them is also nearly identical. Most of multiple domains are two domains, so the length ratio of the first domain to its sequence is close to 0.5. The characteristic of equipartition reflects the folding rules of RNA from a new angle, which is more close to natural folding. Applying this characteristic, algorithm can be designed to dynamically predict long RNA structure, and the dynamic folding mechanism and the relation of function, mutation and RNA structure can be deeply understood from a new view.

## Competing interests

The authors declare that they have no competing interests.

## Declarations

This work was supported by NSFC under grant N0.61070019, 61272431, Shan Dong Province Natural Science Foundation of China under grant N0.ZR2011FL029, ZR2013FM016, the Open Project Program of the Shandong Provincial Key Lab of Software Engineering under grant No.2011SE004, and Program for Scientific Research Innovation Team in Colleges and Universities of Shandong Province.

## Authors' contributions

HL and DM initiated the project and carried out data analysis. KAC conducted statistical modelling and comparison. CZ and HH performed data processing. All authors read and approved the final manuscript.

## References

[B1] Staple DW Butcher SE Pseudoknots: RNA structures with diverse functions PLoSBiol 2005 3 e213 10.1371/journal.pbio.0030213 PMC1149493 15941360

[B2] Mathews DH Turner DH Prediction of RNA secondary structure by free energy minimization Current Opinion in Structural Biology 2006 16 270 278 16713706 10.1016/j.sbi.2006.05.010

[B3] Wiese Kay C Hendriks Andrew Comparison of ***P-RnaPredict ***and ***mfold***--algorithms for RNA secondary structure prediction Bioinformatics 2006 22 934 942 16473869 10.1093/bioinformatics/btl043

[B4] RNA STRAND http://www.rnasoft.ca/strand/

[B5] Bork P Shuffled domains in extracellular proteins FEBSLett 1991 286 47 54 10.1016/0014-5793(91)80937-x 1864378

[B6] Wheelan SJ Marchler-Bauer A Bryant SH Domain size distributions can predict domain boundaries Bioinformatics 2000 16 613 618 11038331 10.1093/bioinformatics/16.7.613

[B7] Petrov AS Bernier CR Hershkovitz E Xue Y Waterbury CC Secondary Structure and Domain Architecture of the 23S rRNA Nucleic Acids Research 2013 41 7522 7535 23771137 10.1093/nar/gkt513 PMC3753638

